# Preparation of rambutan-like Co_0.5_Ni_0.5_Fe_2_O_4_ as anode for high–performance lithium–ion batteries

**DOI:** 10.3389/fchem.2022.1052560

**Published:** 2022-10-20

**Authors:** Qian Wang, Yongzi Wu, Ning Pan, Chenyu Yang, Shuo Wu, Dejie Li, Shaonan Gu, Guowei Zhou, Jinling Chai

**Affiliations:** ^1^ College of Chemistry, Chemical Engineering and Materials Science, Shandong Normal University, Jinan, China; ^2^ Key Laboratory of Fine Chemicals in Universities of Shandong, Jinan Engineering Laboratory for Multi-scale Functional Materials, School of Chemistry and Chemical Engineering, Qilu University of Technology (Shandong Academy of Sciences), Jinan, China

**Keywords:** ternary metal oxides, rambutan-like nanostracture, synergetic effect, anode material, lithium-ion battery

## Abstract

NiFe_2_O_4_ is a kind of promising lithium ion battery (LIB) electrode material, but its commercial applications have been limited due to the electronic insulation property and large volume expansion during the conversion reaction process, which results in rapid capacity decrease and poor cycling stability. We synthesized rambutan-like Co_0.5_Ni_0.5_Fe_2_O_4_ using the self-templating solvothermal method. The special structure of Co_0.5_Ni_0.5_Fe_2_O_4_ which was formed by the assembly of numerous nanosheets could effectively buffer the volume change during the charging and discharging process. Partial substitution of Ni with Co. in NiFe_2_O_4_ leads to Co_0.5_Ni_0.5_Fe_2_O_4_, the coexisting of both nickel and cobalt components is expected to provide more abundant redox reactions. The specific capacity of the rambutan-like Co_0.5_Ni_0.5_Fe_2_O_4_ as an anode material for LIB could reach 963 mA h g^−1^ at the current density of 500 mA g^−1^ after 200 cycles, confirming that the as-synthesized material is a promising candidate for LIBs.

## Introduction

LIB has been widely used in portable electronic items and electric vehicles as an important electronic energy storage device (Huang et al., 2018). With the increasing demand for practical application, developing high electrochemical performance LIB electrode materials is highly desirable (Yang et al., 2017; Luo et al., 2019). Metal oxides have been the focus of recent research on LIB anode materials due to the advantages including high theoretical specific capacity, high power density, pseudo capacitance effect and practical safety (Bi et al., 2020; Lu et al., 2018a; Song et al., 2018; J. Wang et al., 2017). In particular, the transition-metal oxides with spinel structure including MnCo_2_O_4_ (Li et al., 2014), NiCo_2_O_4_ (Shen et al., 2015), and Co_3_O_4_ (Wang et al., 2013) have been the research hotspot of anode materials for LIBs due to the distinctive crystal structure, synergistic reaction between metal ions, multiple oxidation states and other advantages (Jiao et al., 2019; Li J. et al., 2019; Weng et al., 2020). However, the electronic insulation performance and ion transport instability of the spinel transition metal oxides lead to the irreversible capacity and poor cycle stability of the battery, which hinder their application (Wang J. et al., 2016; Wu et al., 2017; Wu et al., 2020).

It was found that inverse spinel structured ferrites can incorporate more Li^+^ than normal spinel structured ferrite (Chen and Greenblatt, 1986). As a typical inverse spinel structured ferrite, NiFe_2_O_4_ has a unique structure in which the Ni^2+^ and half of the Fe^3+^ occupies octahedral positions while the rest Fe^3+^ are distributed in tetrahedral voids (Qu et al., 2017). Therefore, during the insertion and extraction process, the structure can accommodate eight Li^+^ per unit, which resulting in the high theoretical capacity of the NiFe_2_O_4_ (Islam et al., 2017; Tong et al., 2017). Nevertheless, as a promising LIB material, the commercial applications of NiFe_2_O_4_ cannot be realized because of the electronic insulation property and large volume expansion in the conversion reaction, which leads to the fast capacity decrease and poor cycling stability (Xiao et al., 2017). Up to now, many efforts have been conducted to solve this problem. One solution is to designing unique internal structures of NiFe_2_O_4_, such as hollow nanospheres (Gao et al., 2017), yolk-shell structure (Liu et al., 2020) nanofibers (Luo et al., 2014) and so on. Another promising strategy is coupling with highly conductive materials, such as graphene (Shen et al., 2021), carbon-based materials (Mujahid et al., 2019) and so on. However, these methods have some disadvantages, such as complicated preparation procedures, harsh conditions, or reducing energy densities by the coupled component (Huang et al., 2014).

Recently, doping with one or several metal ions to form ternary metal oxide by controlling morphology and composition has become one of the effective methods to modify spinel oxides. For examples, Li et al. synthesized double-shelled Co_0.5_Ni_0.5_MoO_4_ hollow spheres through a facile spray drying process with further post annealing (Li L. et al., 2019), Lu et al. prepared NiCo_2_V_2_O_8_ with yolk-double-shelled structure through a facile self-templating strategy (Lu et al., 2018b). Recently, our group demonstrated that the yolk–double shelled Mn_0.5_Zn_0.5_Co_2_O_4_/C nanospheres could obtain outstanding Li-ions storage performance (Ren et al., 2022a). The several possible redox sites, multiple oxidation states and abundant active sites in the ternary metal systems could help to increase the charge diffusion rate and ion-diffusion rate, which further improves the electronic conductivity, electrochemical activity, mechanical and chemical stability (Gonçalves et al., 2020).

Based on above researches, doping a multi-chemical valence ions, such as cobalt ions, into the lattice of inverse spinel NiFe_2_O_4_ may be an effective method to improve the lithium storage ability of NiFe_2_O_4_ through enhancing the electronic conductivity due to the synergistic effect between the several metals (Li J. et al., 2019). In addition, the combination of nickel and cobalt species is helpful to offer more abundant redox reactions (Liang et al., 2014; Guan et al., 2017). Furthermore, we synthesized rambutan-like Co_0.5_Ni_0.5_Fe_2_O_4_ using the self-templating solvothermal method. The special structure of Co_0.5_Ni_0.5_Fe_2_O_4_ which was formed by the assembly of numerous nanosheets could effectively buffer the volume change during the charging and discharging process. Partial substitution of Ni with Co. in NiFe_2_O_4_ leads to Co_0.5_Ni_0.5_Fe_2_O_4_, which is a promising material that integrates the high capacity of NiFe_2_O_4_ and the cycling stability of CoFe_2_O_4_ (Li L. et al., 2019). The specific capacity of the rambutan-like Co_0.5_Ni_0.5_Fe_2_O_4_ as a LIB anode material could reach 963 mA h g^−1^ at the current density of 500 mA g^−1^ after 200 cycles, confirming the as-synthesized material is a potential candidate for LIBs.

## Experimental section

### Chemicals

Fe(NO_3_)_3_·9H_2_O, Ni(NO_3_)_2_·6H_2_O and Co.(NO_3_)_2_·6H_2_O were purchased from Sigma-Aldrich. Isopropyl alcohol, glycerol and ethanol were purchased from Sinopharm Chemical Reagent Co., Ltd. All purchased chemicals were directly used. Ultrapure water used throughout the experiments was obtained from a MilliQ water purification system.

### Synthesis of Co_0.5_Ni_0.5_Fe_2_O_4_

Firstly, 0.101 g of Fe(NO_3_)_3_·9H_2_O, 0.0182 g of Co.(NO_3_)_2_·6H_2_O and 0.0183 g of Ni(NO_3_)_2_·6H_2_O were added to 48 ml mixed solvents of glycerol and isopropanol (1:5 by volume) under stirring. The formed orange-yellow clear solution was poured into a 100 ml Teflon container, then heated to 180°C and reacted for 6 h. The product was washed by centrifugation with ethanol and then dried at 60°C. Finally, the obtained yellow precipitate was annealed at 400°C in air for 2 h to form Co_0.5_Ni_0.5_Fe_2_O_4_ nanospheres. The NiFe_2_O_4_ and CoFe_2_O_4_ were synthesized through the same method.

### Structural characterization

X-ray diffraction (XRD) patterns were obtained using a Bruker D8-ADVANCE (40 kV, 40 mA) diffractometer. The morphology and structure of the products were observed using field emission electron microscope (FESEM, Hitachi S-4800) and high resolution transmission electron microscope (HRTEM, JEM-2100). The elemental valence was analyzed through X-ray photoelectron spectroscope which was collected by the X-ray energy spectrometer (ESCALAB MKII). The surface area and porosity analyzer (ASAP 2020 HD88) was used to analyze the specific surface area and pore size distribution. The accurate content of Co., Ni and Fe was analyzed by ICP-MS (NexION 350X).

### Electrodes fabrication and electrochemical measurements

The prepared samples, acetylene black, and sodium carboxymethyl cellulose (CMC, 10 wt%) are mixed together with the mass ratio of 7:2:1. After adding solvent deionized water, the mixture was uniformly ground for 3 h using a planetary ball mill, then coated on the Cu foil. The coated Cu foil was cut into circular slices with the radius of 6 mm after drying in vacuum, and each with the area loading of about 1.5 mg cm^−2^. In a glovebox filled with argon, coin cell was prepared using the Celgard 2,400 as separator, and the lithium metal foil as counter electrode. The electrolyte is 40 µl of 1.0 mol L^−1^ LiPF_6_ dissolved in a 1:1:1 volume ratio of EC, DMC, and EMC. The LAND electrochemical station was used to test the cycling performance and rate capability of the cells. The PARSTAT 4000 electrochemical workstation was used to collect Cyclic voltammogram (CV) and electrochemical impedance spectra (EIS).

## Results and discussion

**SCHEME 1:**
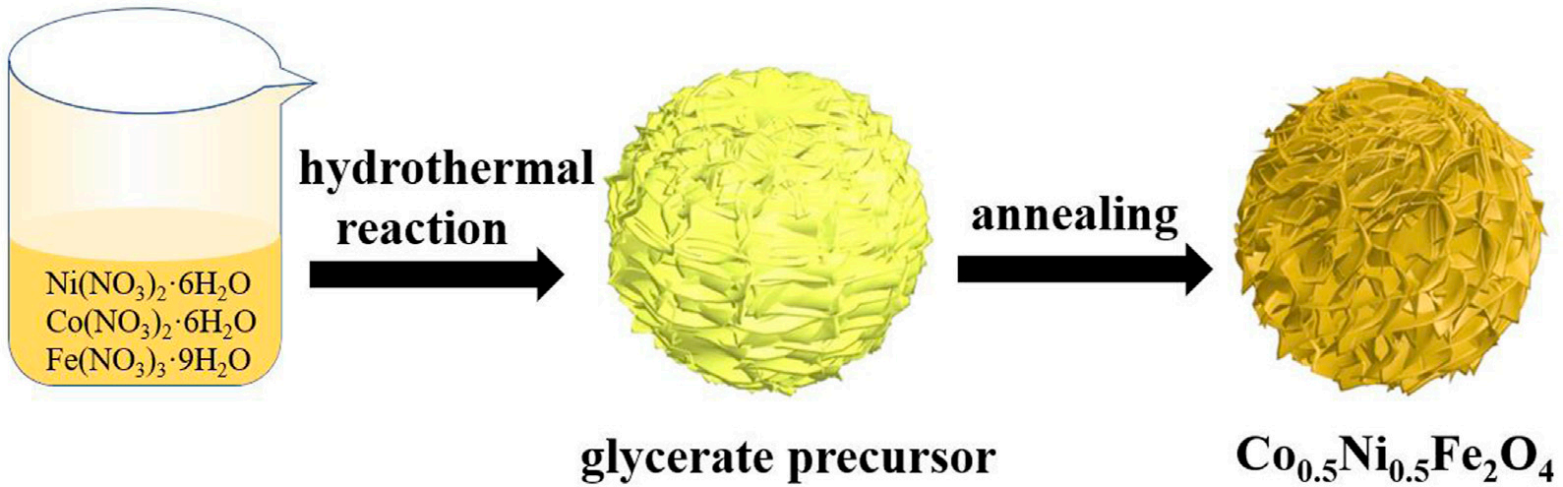
Schematic diagram of the preparation process of Co_0.5_Ni_0.5_Fe_2_O_4_ nanospheres.

The strategy and mechanism for preparing Co_0.5_Ni_0.5_Fe_2_O_4_ nanospheres is illustrated in Scheme 1. Firstly, CoNiFe-glycerate was prepared through the solvothermal method. The Ni^2+^, Co^2+^ and Fe^3+^ precipitated as Co-Ni-Fe hydroxides with the OH^−^ released by the redox reaction of isopropanol and NO^3−^, resulting in the formation of uniform CoNiFe-glycerate nanospheres (Supplementary Figure S1). The rambutan-like structure was gradually formed in the process of solvent heat treatment due to the dehydration condensation reaction of the hydroxyl groups in glycerol at high temperature. Lastly, the CoNiFe-glycerate nanospheres transformed into Co_0.5_Ni_0.5_Fe_2_O_4_ nanospheres through simple heat treatment.

**FIGURE 1:**
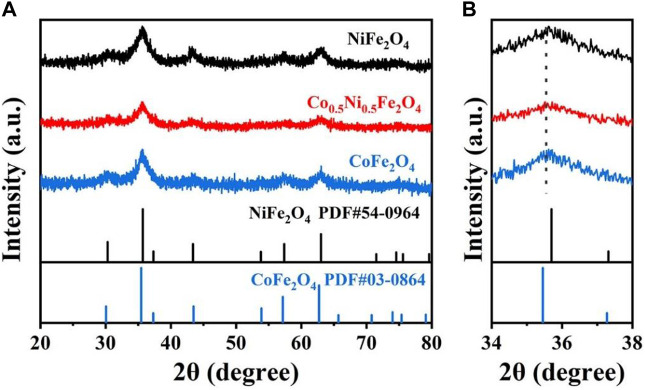
**(A)** XRD patterns of the obtained Co_0.5_Ni_0.5_Fe_2_O_4_, NiFe_2_O_4_ and CoFe_2_O_4_. **(B)** XRD comparison of Co_0.5_Ni_0.5_Fe_2_O_4_, NiFe_2_O_4_ and CoFe_2_O_4_ at 34°–38° after magnification.

Figure 1A shows XRD patterns of the synthesized Co_0.5_Ni_0.5_Fe_2_O_4_, NiFe_2_O_4_ and CoFe_2_O_4_ materials. The synthesized NiFe_2_O_4_ and CoFe_2_O_4_ corresponded to NiFe_2_O_4_ (JCPDS No. 54-0964) and CoFe_2_O_4_ (JCPDS No. 03-0864), respectively. The characteristic peak of Co_0.5_Ni_0.5_Fe_2_O_4_ was quite similar to NiFe_2_O_4_ and CoFe_2_O_4_ without extra lines representing the Co_0.5_Ni_0.5_Fe_2_O_4_ has a single-phase and shares the same structure with both NiFe_2_O_4_ and CoFe_2_O_4_. The characteristic (311) plane at 2θ of 35.5 also indicating the spinel structure of Co_0.5_Ni_0.5_Fe_2_O_4_ sample (Shehnaz et al., 2021). The slight peak shift can be observed in Figure 1B, and the characteristic peak of Co_0.5_Ni_0.5_Fe_2_O_4_ was found between NiFe_2_O_4_ and CoFe_2_O_4_. Indirectly, the successful fabrication of the ternary metal oxide Co_0.5_Ni_0.5_Fe_2_O_4_ was demonstrated. The shift of peak position implying the expansion of lattice volume which is attributed to the different ionic radii of Ni^2+^ (0.69 Å) and Co^2+^ (0.74 Å) ions (Chen et al., 2015).

**FIGURE 2:**
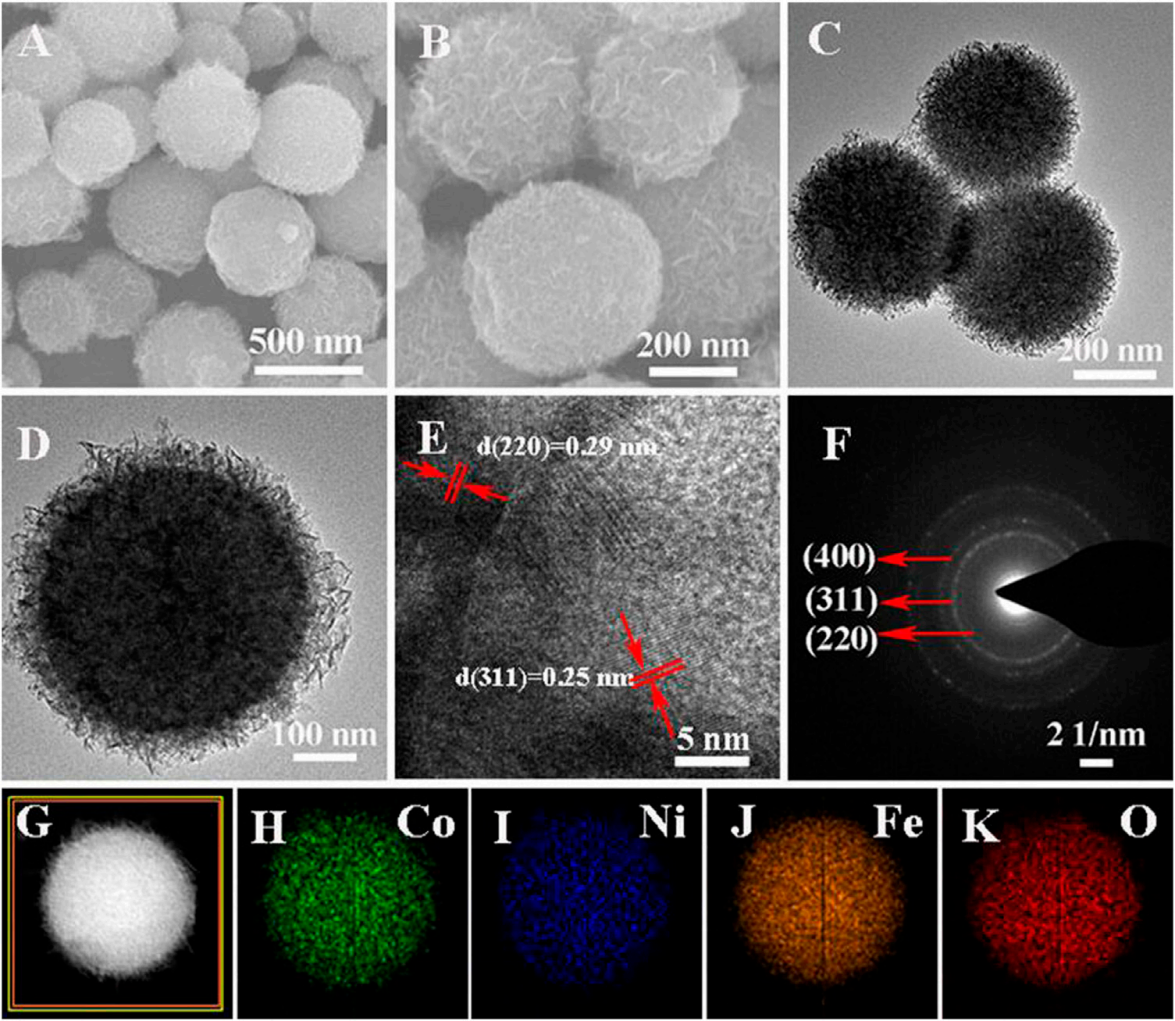
**(A–B)** SEM images of Co_0.5_Ni_0.5_Fe_2_O_4_ **(C–D)** TEM images of Co_0.5_Ni_0.5_Fe_2_O_4_; **(E)** HRTEM images of Co_0.5_Ni_0.5_Fe_2_O_4_ **(F)** SAED patterns of Co_0.5_Ni_0.5_Fe_2_O_4_; **(G–K)** EDX element mappings of Co_0.5_Ni_0.5_Fe_2_O_4_.

The SEM images (Figures 2A,B) show that the Co_0.5_Ni_0.5_Fe_2_O_4_ were highly uniform spheres of about 500 nm in size. As shown in Figures 2C,D, the Co_0.5_Ni_0.5_Fe_2_O_4_ possessed a rambutan-like structure. The SEM and TEM images of NiFe_2_O_4_ and CoFe_2_O_4_ were shown in Supplementary Figure S1, both the NiFe_2_O_4_ and CoFe_2_O_4_ were highly uniform spheres of about 500 nm in size, which were similar to the Co_0.5_Ni_0.5_Fe_2_O_4_. Figure 2E shows the HRTEM image of a single Co_0.5_Ni_0.5_Fe_2_O_4_, the lattice spacings of 0.25, 0.29 and 0.48 nm identical to (311) (220) and (111) d-spacing of Co_0.5_Ni_0.5_Fe_2_O_4_, respectively. Meanwhile, three corresponding rings in the crystal plane were observed clearly in the selected area electron diffraction (SAED) image (Figure 2F) indicating that the crystallinity of the Co_0.5_Ni_0.5_Fe_2_O_4_ is good. In addition, the EDS element mapping images of Co., Ni, Fe, O (Figures 2G–K) indicating the homogeneous distribution of these elements in Co_0.5_Ni_0.5_Fe_2_O_4_ and further indicating the single-phase property of the obtained products. To confirm the chemical composition of the Co_0.5_Ni_0.5_Fe_2_O_4_, the Co./Ni value was identified by ICP–MS, which was nearly 1.15:1, approach to the feeding ratio (1: 1).

**FIGURE 3:**
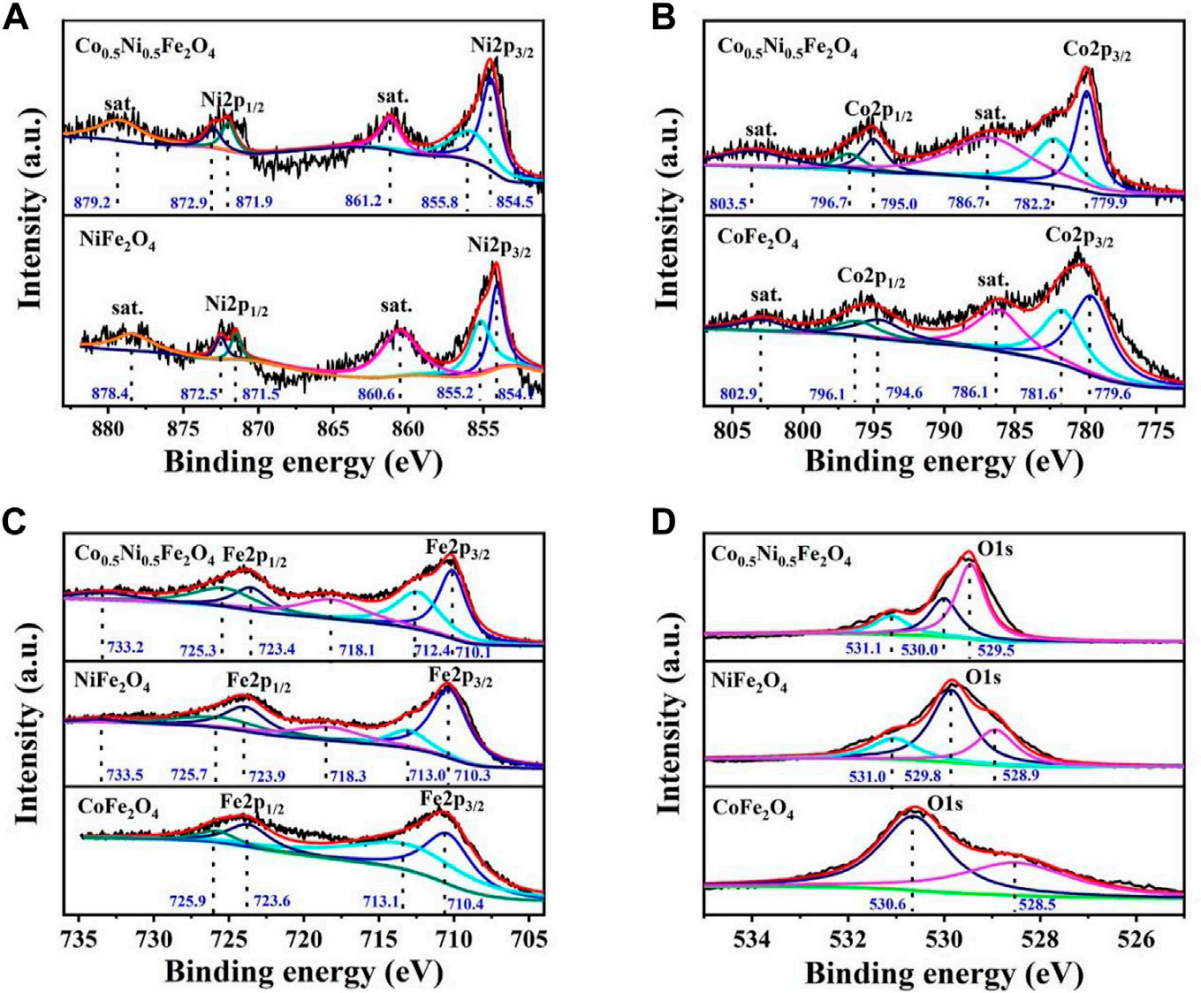
XPS spectra of the Co_0.5_Ni_0.5_Fe_2_O_4_, NiFe_2_O_4_ and CoFe_2_O_4_ spheres **(A)** Ni 2p; **(B)** Co. 2p **(C)** Fe 2p; and **(D)** O1s.

To further illustrate the chemical composition of Co_0.5_Ni_0.5_Fe_2_O_4_, XPS characterizations were employed to analyze the valence states of each element in the Co_0.5_Ni_0.5_Fe_2_O_4_, NiFe_2_O_4_ and CoFe_2_O_4_ spheres, respectively. Figure 3A shows the Ni 2 p spectrum, the characteristic peaks centered at 854.5 and 871.9 eV are attributed to Ni^2+^ species while the peaks located at 855.8 and 872.9 eV represent the Ni^3+^ ion. Compared with NiFe_2_O_4_, the peak location of Co_0.5_Ni_0.5_Fe_2_O_4_ moved 0.4–0.8 eV to high binding energy, which attributed to the charge transfer as well as the strong interaction of the Ni–O bond. The Co_2p_ peaks at about 779.9 and 795.0 eV (Figure 3B) are attributed to Co^2+^ and the peaks at 782.2 and 796.7 eV indicating the presence of Co^3+^, which is the result of the partial oxidation of Co^2+^ from the surface (Shehnaz et al., 2021). Meanwhile, because of the strong Co–O interaction, the binding energy of Co_0.5_Ni_0.5_Fe_2_O_4_ increased 0.3–0.6 eV than that of CoFe_2_O_4_. The 2p_3/2_ peaks at about 710.1and 712.4 eV (Figure 3C), along with the 2p_1/2_ peaks at around 723.4 and 725.3 eV represents the coexist of both the tetrahedral site and octahedral sites Fe^3+^ species. The satellite peaks at around 718.1 eV and 733.2 eV, also represent the presence of Fe^3+^ cations (Shehnaz et al., 2021). Compared to NiFe_2_O_4_ and CoFe_2_O_4_, the binding energy of Ni 2 p and Co. 2 p in Co_0.5_Ni_0.5_Fe_2_O_4_ were increased while that of Fe 2 p were decreased, which means the electrons transfer from Ni and Co. to Fe. The shift of the binding energy further demonstrated the prepared Co_0.5_Ni_0.5_Fe_2_O_4_ was ternary metal oxide. The characteristic peaks of Co_0.5_Ni_0.5_Fe_2_O_4_ at 529.5, 529.8 and 531.0 eV (Figure 3D) indicating the metal oxygen, oxygen vacancy and adsorption oxygen, respectively. The binding energy of the oxygen vacancy for Co_0.5_Ni_0.5_Fe_2_O_4_, NiFe_2_O_4_ and CoFe_2_O_4_ were 530.0, 529.8 and 530.6 eV (Kang et al., 2019; Pramanik et al., 2020; Ren et al., 2022b), respectively. The binding energy of O1s changes obviously with the doping of Co., indicating the successfully synthesized of Co_0.5_Ni_0.5_Fe_2_O_4_.

The nitrogen adsorption-desorption experiment was conducted to test the specific surface area and pore size distribution of the three samples. As can be seen in Supplementary Figure S2, all three samples have obvious hysteresis loops, indicating they are all mesoporous structure (Zhou et al., 2017). According to the analyzed results, the BET surface areas were 242.63, 177.43 and 146.84 m^2^ g^−1^ for Co_0.5_Ni_0.5_Fe_2_O_4_, NiFe_2_O_4_ and CoFe_2_O_4,_ while the corresponding average diameter were 3.1, 3.2 and 4.05 nm, respectively. The higher specific surface area of Co_0.5_Ni_0.5_Fe_2_O_4_ is attributed to the greater surface roughness than NiFe_2_O_4_ and CoFe_2_O_4_ spheres which can be seen from the TEM and SEM images. Because of the larger BET surface area and smaller average pore size, the Co_0.5_Ni_0.5_Fe_2_O_4_ can offer more active sites, shorten the Li^+^ transport path, and help to the reduction of polarization in the process of charge and discharge (Qu et al., 2017).

**FIGURE 4:**
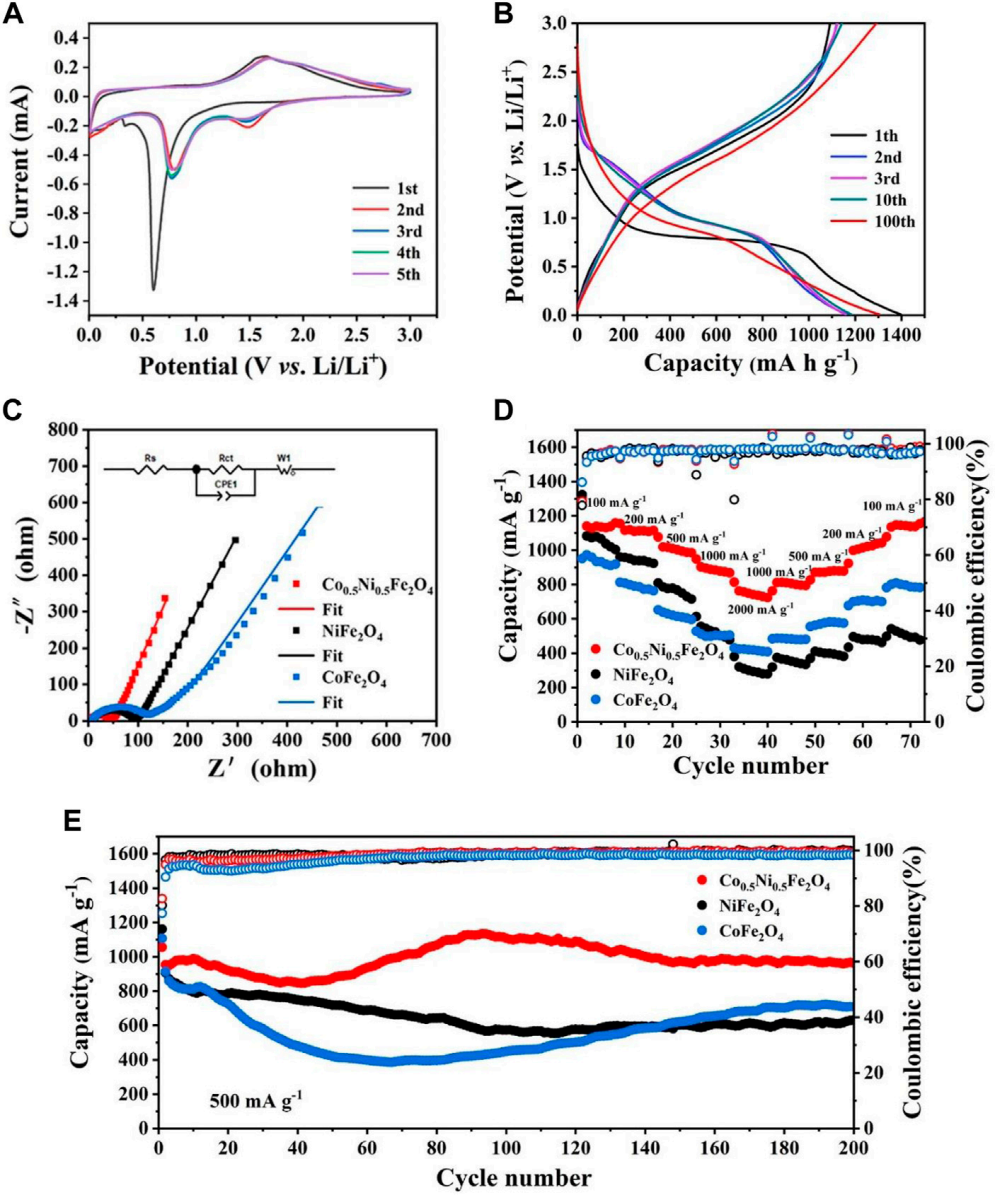
**(A)** CV curves of Co_0.5_Ni_0.5_Fe_2_O_4_ in the first five cycles at 0.1 mV s^−1^. **(B)** Discharge-charge curves of Co_0.5_Ni_0.5_Fe_2_O_4_ at 200 mA g^−1^. **(C)** Nyquist plots of the Co_0.5_Ni_0.5_Fe_2_O_4_, NiFe_2_O_4_ and CoFe_2_O_4_. **(D)** Rate capability of Co_0.5_Ni_0.5_Fe_2_O_4_, NiFe_2_O_4_ and CoFe_2_O_4_. **(E)** Cycling performance and the corresponding Coulombic efficiencies of Co_0.5_Ni_0.5_Fe_2_O_4_, NiFe_2_O_4_ and CoFe_2_O_4_ at 500 mA g^−1^.

Figure 4A shows the CV curves of Co_0.5_Ni_0.5_Fe_2_O_4_ at 0.1 mV s^−1^ between 0.01 and 3.00 V. In the first cycle, two cathodic peaks at around 0.3 and 0.6 V were attributed to the reduction of Co., Ni and Fe in Co_0.5_Ni_0.5_Fe_2_O_4_ (Eq. 1) with the simultaneous formation of both Li_2_O and SEI layer, one anode peak located at 1.6 V was corresponded to the oxidation of Ni, Co., and Fe (Ding et al., 2013). The cathodic peak shifted from 0.6 to about 0.8 V in the following four cycles, with a new peak appears around 1.5 V simultaneously which indicating the reversible reduction of Fe_2_O_3_ and MeO, respectively (Xia et al., 2021). The anode peak at 1.6 V moved to 1.7 V which was caused by the polarization of the battery (Xiao et al., 2017). The electrode reaction of Co_0.5_Ni_0.5_Fe_2_O_4_ can be expressed by the following equation:

$$2{Co}_{0.5}{Ni}_{0.5}{Fe}_{2}O_{4}+16e^{–}+16{Li}^{+}\to Co+Ni+4Fe+8{Li}_{2}O$$ (1)

$$Ni+{Li}_{2}O\to NiO+2{Li}^{+}+2e^{–}$$ (2)

$$Co+{Li}_{2}O\to CoO+2{Li}^{+}+2e^{–}$$ (3)

$$2Fe+3{Li}_{2}O\to {Fe}_{2}O_{3}+6{Li}^{+}+6e^{–}$$ (4)

The nearly overlapped CV curves and characteristic peaks after the first cycle, implying the electrode had satisfied reversibility and cyclic stability during discharge and charge process (Zhang et al., 2019a). The cyclic voltammograms of NiFe_2_O_4_ and CoFe_2_O_4_ are shown in Supplementary Figure S3A and S4A, and the similar profiles illustrating they have approximate lithium storage mechanism with Co_0.5_Ni_0.5_Fe_2_O_4_. The CV curves of the three samples were slightly different, which was attributed to their different chemical compositions. The charge-discharge curve at 200 mA g^−1^ between 0.01 and 3 V is shown in Figure 4B. The discharge plateau of the first cycle was around 0.6 V and increased to 0.8 V subsequently due to the irreversible reaction, which are identical to the CV curve. The initial discharge and charge capacity of Co_0.5_Ni_0.5_Fe_2_O_4_ are 1397.17 and 1092.01 mA h g^−1^, respectively. The initial Coulombic efficiency (CE) is 78.16%, and the capacity loss of 21.84% is ascribed to the irreversible reaction and SEI film decomposition (Zhang et al., 2019b; Zou et al., 2020). Due to the activation of the electrode, the discharge capacity of the Co_0.5_Ni_0.5_Fe_2_O_4_ electrode decreased at the early stage, but surprisingly, the specific capacity could maintain at 1304.5 mA h g^−1^ after 100 cycles, which is obviously higher than those of NiFe_2_O_4_ (644 mA h g^−1^) and CoFe_2_O_4_ (738 mA h g^−1^) as shown in Supplementary Figure S5.

Electrochemical impedance spectroscope (EIS) was performed to testing the ability of electrode materials participating in the chemical reaction. The semicircle of the Nyquist diagram in the high frequency region represents charge transfer impedance (*R*_ct_) on the interface of electrode and electrolyte, and the sloped line of Nyquist diagram in the low frequency region correspond to ion diffusion resistance in the electrodes (Gong et al., 2018). Figure 4C shows the Nyquist plots of Co_0.5_Ni_0.5_Fe_2_O_4_, NiFe_2_O_4_ and CoFe_2_O_4_. According to the fitting results, the *R*_ct_ of Co_0.5_Ni_0.5_Fe_2_O_4_ was about 36.6 Ω while the values of NiFe_2_O_4_ and CoFe_2_O_4_ were 77.7 and 86 Ω, respectively. The EIS spectrum of Co_0.5_Ni_0.5_Fe_2_O_4_ also exhibits larger slope than those of NiFe_2_O_4_ and CoFe_2_O_4_ in the low frequency regime. The EIS results shows that Co_0.5_Ni_0.5_Fe_2_O_4_ anode material has lower resistance, which is contributed to the transfer of electron and Li^+^, resulting in the improved electrochemical performance.

Figure 4D depicts the rate capability of the three electrode materials. When the current density was up to 2000 mA g^−1^, the capacity of Co_0.5_Ni_0.5_Fe_2_O_4_ can reach 763 mA h g^−1^. As the current density gradually returned to 100 mA g^−1^, the specific capacity of Co_0.5_Ni_0.5_Fe_2_O_4_ increased to 1154 mA h g^−1^ while the corresponding CE maintained at around 98.9%. This result indicated that the cycling stability of Co_0.5_Ni_0.5_Fe_2_O_4_ is very well. However, as the current density decreased gradually, the specific capacity of NiFe_2_O_4_ did not increase obviously, indicating its cyclic stability was poor. Although the capacity of CoFe_2_O_4_ increased as the current density decreased, the capacity only reached 785 mA h g^−1^ at 100 mA g^−1^. The results indicating that the Co_0.5_Ni_0.5_Fe_2_O_4_ has significantly higher specific capacity and stability than NiFe_2_O_4_ and CoFe_2_O_4_.

Figure 4F shows the results of cyclic stability of the samples at 500 mA g^−1^ for 200 cycles. The specific capacity of NiFe_2_O_4_ gradually decreased during the initial 90 cycles, then maintained at about 631.5 mA h g^−1^ as the number of cycles increases. During the first 60 cycles, the specific capacity of CoFe_2_O_4_ decreased to 400 mA h g^−1^, and then increased to 708.5 mA h g^−1^ after 200 cycles. In contrast, the capacity of Co_0.5_Ni_0.5_Fe_2_O_4_ has a slightly decrease in the first 40 cycles, then gradually increases and finally stabilizes at about 963 mA h g^−1^ after 200 cycles. The specific capacity of the Co_0.5_Ni_0.5_Fe_2_O_4_ electrode increases with the number of cycles, and then slightly decreases with the fluctuation, which might be originated from the much slower and more sufficient activation of the fresh electrode, as well as the decreased side reactions in the electrode/electrolyte interface after the formation of SEI (Wang Z. et al., 2016; Gao et al., 2017). In addition, the increase in specific capacity may be related to factors such as activation and adequate infiltration of the electrolyte, as depicted and described in many other publications (Xu et al., 2020; Zhang et al., 2020). The initial Coulombic Efficiency (CE) of Co_0.5_Ni_0.5_Fe_2_O_4_ electrode was 82.7% at the current density of 500 mA g^−1^. The long cycle curves of samples at the current densities of 200 and 1000 mA g^−1^ are shown in Supplementary Figure S6. When cycled at the current densities of 200 and 1000 mA g^−1^, the initial CEs of the Co_0.5_Ni_0.5_Fe_2_O_4_ electrodes were determined to be 78.2% and 79.3%, respectively. The capacity of Co_0.5_Ni_0.5_Fe_2_O_4_ can stabilized at 1190 mA h g^−1^ after 200 cycles at 200 mA g^−1^. When the current density reached 1000 mA g^−1^, the specific capacity of both the NiFe_2_O_4_ and CoFe_2_O_4_ decreased rapidly and finally stabilized at about 400 mA h g^−1^. However, the capacity of Co_0.5_Ni_0.5_Fe_2_O_4_ has a slightly decrease in the first 60 cycles at 1000 mA g^−1^, then increased gradually and tend to be stable, finally stabilized at about 850 mA h g^−1^ after 200 cycles. This result demonstrated the superior electrochemical stability of the Co_0.5_Ni_0.5_Fe_2_O_4_.

**FIGURE 5:**
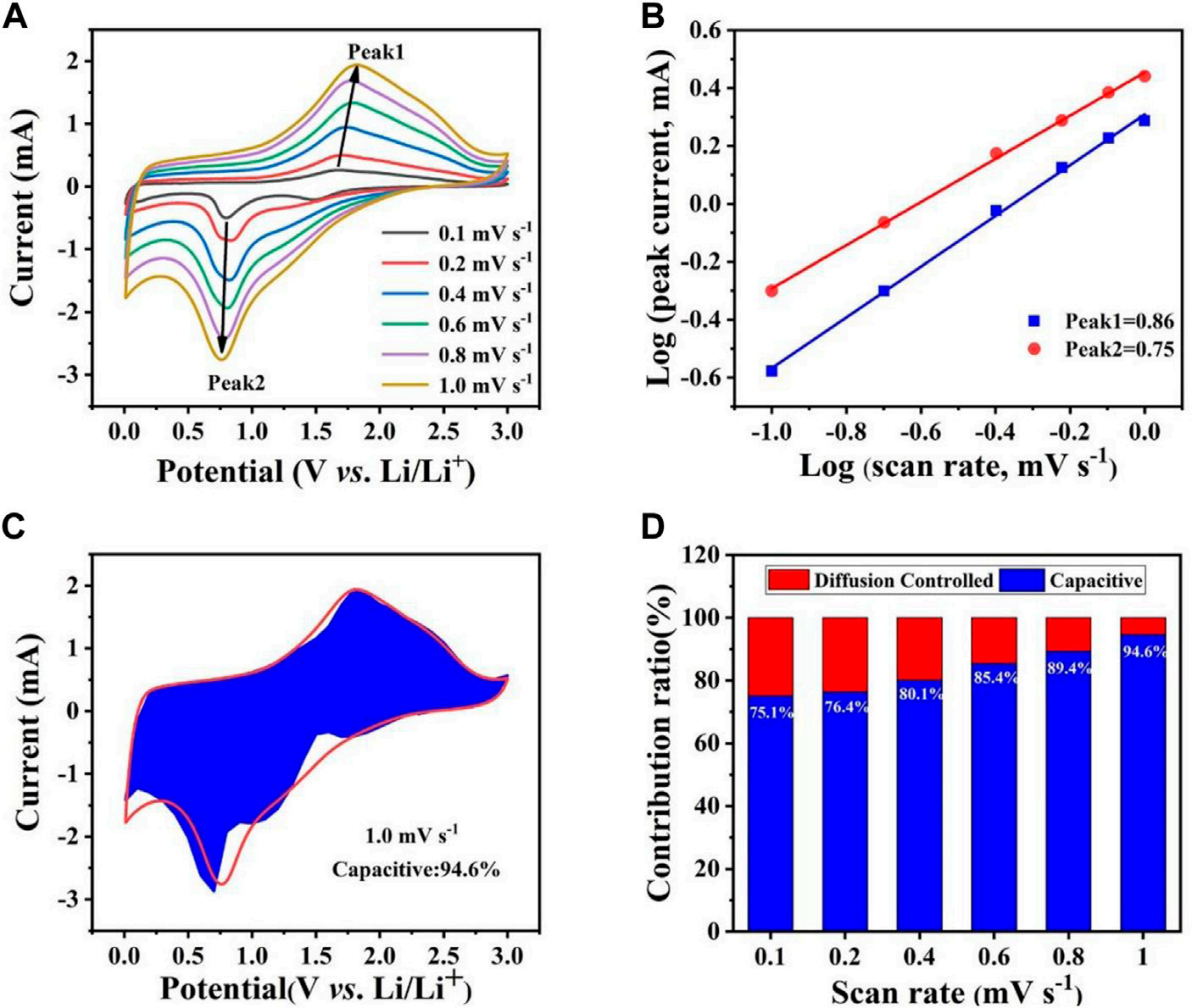
**(A)** CV curves of Co_0.5_Ni_0.5_Fe_2_O_4_ at various scan rates ranging from 0.1 mV s^−1^ to 1.0 mV s^−1^. **(B)** the relationship between log(i) and log(*v*). **(C)** The capacitive contribution of Co_0.5_Ni_0.5_Fe_2_O_4_ at 1.0 mV s^−1^. **(D)** Pseudocapacitive contribution of Co_0.5_Ni_0.5_Fe_2_O_4_ at different scan rates.

The CV test was performed (Figure 5A) to further explore the lithium storage mechanism of the Co_0.5_Ni_0.5_Fe_2_O_4_, which can be calculated using the equation:

log(i)=b⁡log(v)+log(a) (5)

Generally, when *b* = 0.5, the electrochemical process contributes by diffusion whereas the capacitive effect will play the dominate role when the value of *b* is up to 1.0 (Jiang and Liu, 2019; Acharya et al., 2021). As shown in Figure 5B, the values of *b* shown by peak one and peak two were 0.75 and 0.86, respectively. This result suggests that the pseudo capacitive effect is the main factor driving the electrochemical process. Figures 5C,D demonstrate that the contribution of the capacitive effect gradually becomes higher as scan rates increase. The contribution of pseudo capacitive for the Co_0.5_Ni_0.5_Fe_2_O_4_ anode reached 94.6% at the scan sweep rate of 1.0 mV s^−1^ while those of the NiFe_2_O_4_ and CoFe_2_O_4_ were 92.0% (Supplementary Figure S3C) and 91.7% (Supplementary Figure S4C), respectively. The result shows that the Co_0.5_Ni_0.5_Fe_2_O_4_ material with higher capacitance contribution can provide more active sites and transmission routes, further improve the electrochemical cycling performance.

**FIGURE 6:**
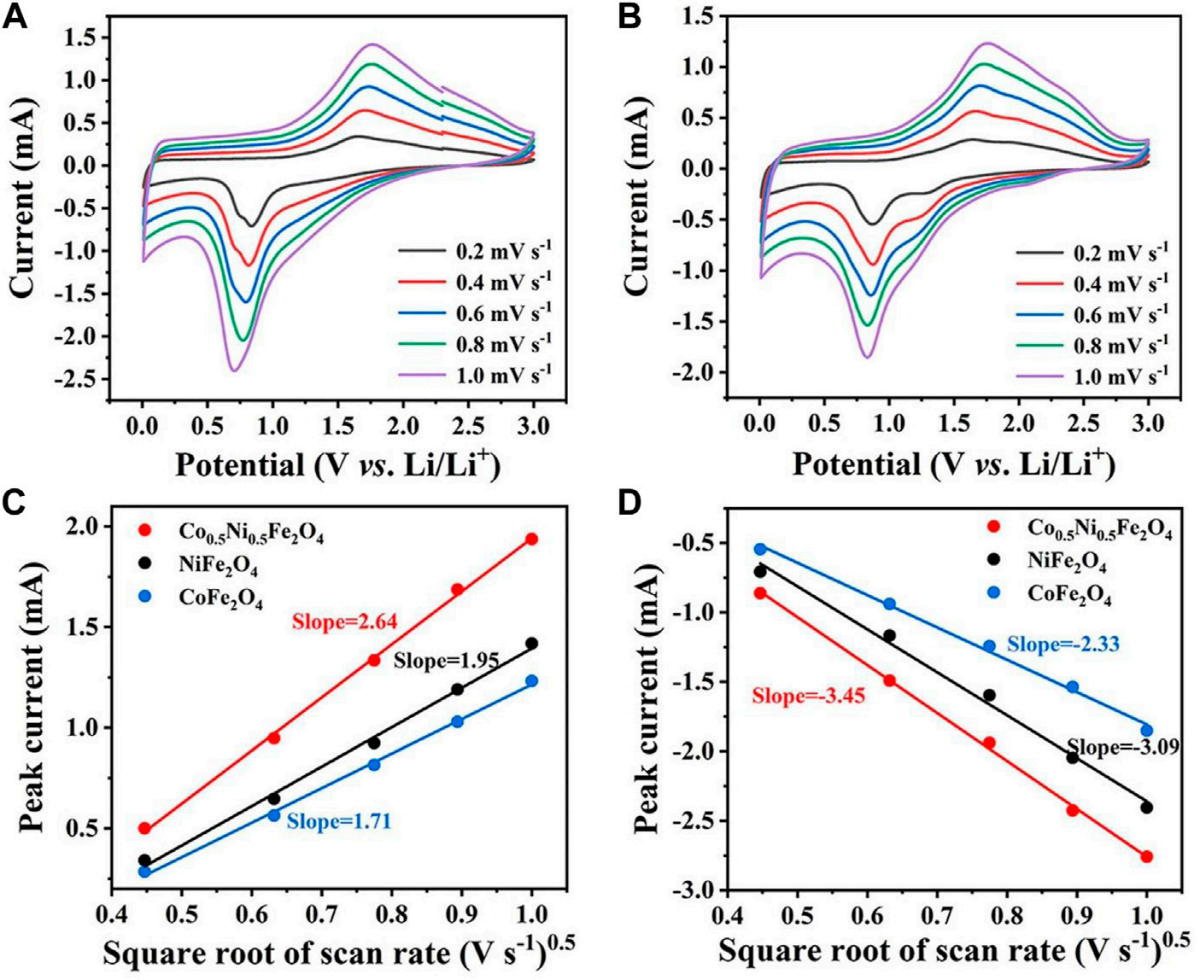
CV curves of **(A)** NiFe_2_O_4_ and **(B)** CoFe_2_O_4_ at various scan rates. Linear relationship between CV peak current of **(C)** anodic and **(D)** cathodic peaks and the square root of scan rate for NiFe_2_O_4_ and CoFe_2_O_4_, and Co_0.5_Ni_0.5_Fe_2_O_4_.

The CV curves of NiFe_2_O_4_ and CoFe_2_O_4_ at different scan rates are shown in Figures 6A,B. The Randles-Sevcik equation can be used to calculate the diffusion coefficient of Li^+^:

$$I_{p}=2.69\times 10^{5}n^{3/2}AD_{Li}^{1/2}v^{1/2}∆C_{0}$$ (6)

*I*_p_ refers to the peak current of the CV curves, *A* represents the electrode area, *n* means the number of electron transfer, *v* is the scan rate, *D* refer to the Li^+^ diffusion coefficient, and *C*_0_ is the concentration of electrolyte. Since *A*, *n*, *v*, and ∆*C*_0_ are all constants in this equation, the slope of the curve can represent the Li^+^-diffusion capability (Wang et al., 2020; Ren et al., 2022a). As shown in Figures 6C,D, the slope of the curve for Co_0.5_Ni_0.5_Fe_2_O_4_ was obviously higher than those of NiFe_2_O_4_ and CoFe_2_O_4_, indicating that the Co_0.5_Ni_0.5_Fe_2_O_4_ has the largest Li^+^ diffusion coefficient which further proved that the rambutan-like sphere with a high surface area was more helpful to the Li^+^ transport.

## Conclusion

In conclusion, we synthesized the rambutan-like ternary metal oxide Co_0.5_Ni_0.5_Fe_2_O_4_ by a facile self-templating solvothermal method. The special structure and large specific surface area of the sample can effectively buffer the volume changes and offer more active sites during the Li^+^ insertion and extraction processes. Due to the synergistic interaction between metal ions, the anode can still maintain a high specific capacity after 200 cycles at the current density of 500 mA g^−1^, indicating the electrode material has strong lithium storage performance. The work shows that the electrochemical performance can be improved through designing unique components and structures of the anode materials. Furthermore, this method can be expanded to prepare additional metal oxide for the fabrication of potential anodes for LIBs.

## Data Availability

The original contributions presented in the study are included in the article/Supplementary Material, further inquiries can be directed to the corresponding authors.
