# Supplementary material for: Preparation of rambutan-like Co0.5Ni0.5Fe2O4 as anode for high–performance lithium–ion batteries
Source: Front Chem. 2022 Oct 20;10:1052560. doi: 10.3389/fchem.2022.1052560 (PMC9631019; doi:10.3389/fchem.2022.1052560)
Supplement: Supplementary file 1 [file DataSheet1.PDF]

# Supplementary Materials

# Preparation of Rambutan-like $Co_{0.5}Ni_{0.5}Fe_2O_4$ as Anode for High–performance Lithium–ion Batteries


Qian Wang[1], Yongzi Wu[2], Ning Pan[1], Chenyu Yang[2], Shuo Wu[2], Dejie Li[1], Shaonan Gu[2*], Guowei Zhou[2*], Jinling Chai[1*]

[1] College of Chemistry, Chemical Engineering and Materials Science, Shandong Normal University, Jinan, 250014, P. R. China

[2] Key Laboratory of Fine Chemicals in Universities of Shandong, Jinan Engineering Laboratory for Multi-scale Functional Materials, School of Chemistry and Chemical Engineering, Qilu University of Technology (Shandong Academy of Sciences), Jinan, 250353, P. R. China

[*] **Corresponding authors:** Tel: +86 531 89631696.

*E-mail addresses*: sngu@qlu.edu.cn (S.N. Gu), gwzhou@qlu.edu.cn (G.W. Zhou), jlchai@sdnu.edu.cn (J.L. Chai)

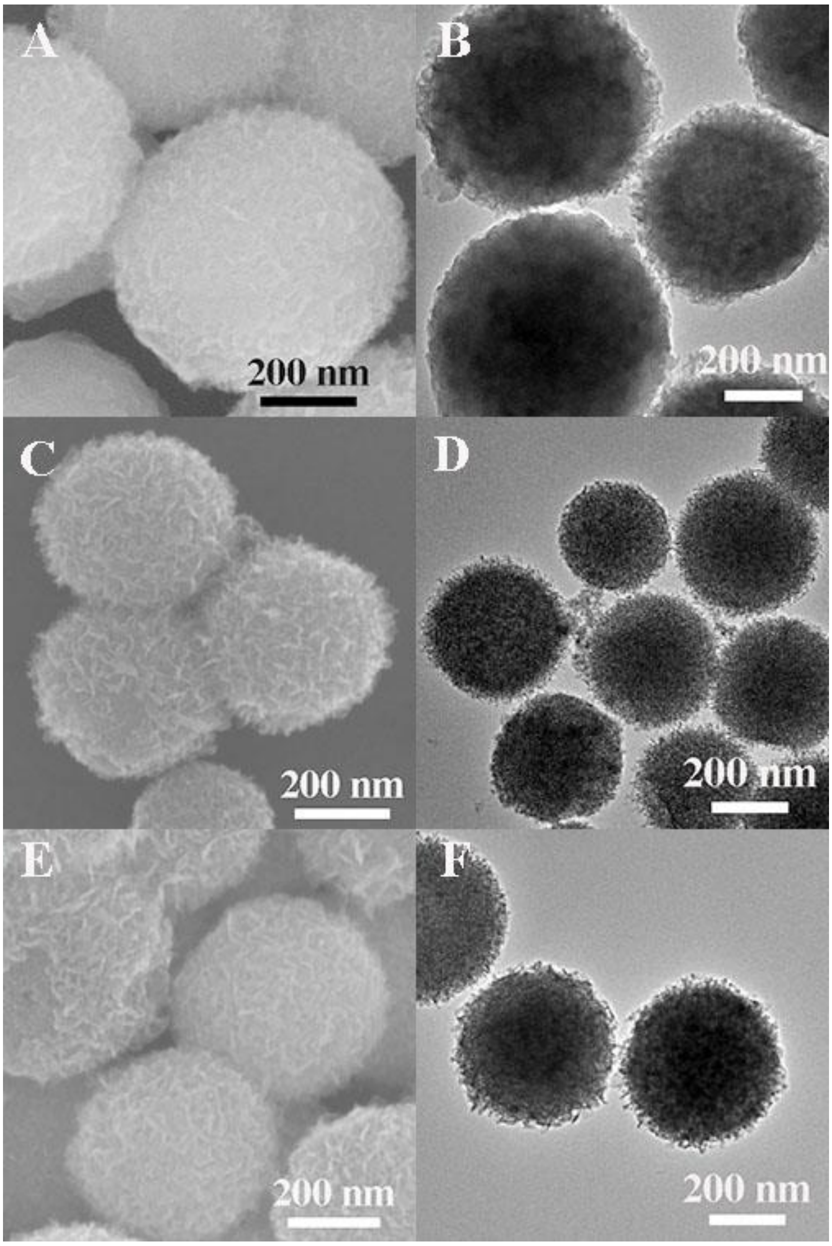


Figure S1. SEM images of the (A) CoNiFe-glycerate nanospheres, (C)$NiFe_2O_4$ nanospheres and (E) $CoFe_2O_4$ nanospheres; TEM images of the(B) CoNiFe-glycerate nanospheres, (D)$NiFe_2O_4$ nanospheres and (F) $CoFe_2O_4$ nanospheres.

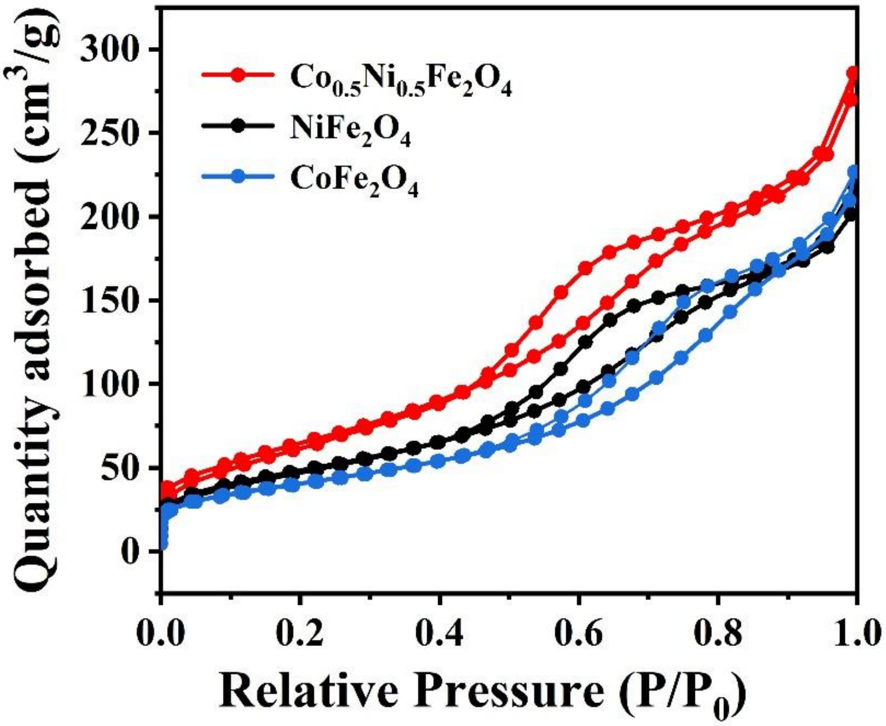


Figure S2. $N_2$ adsorption–desorption plots of the $Co_{0.5}Ni_{0.5}Fe_2O_4$ spheres.

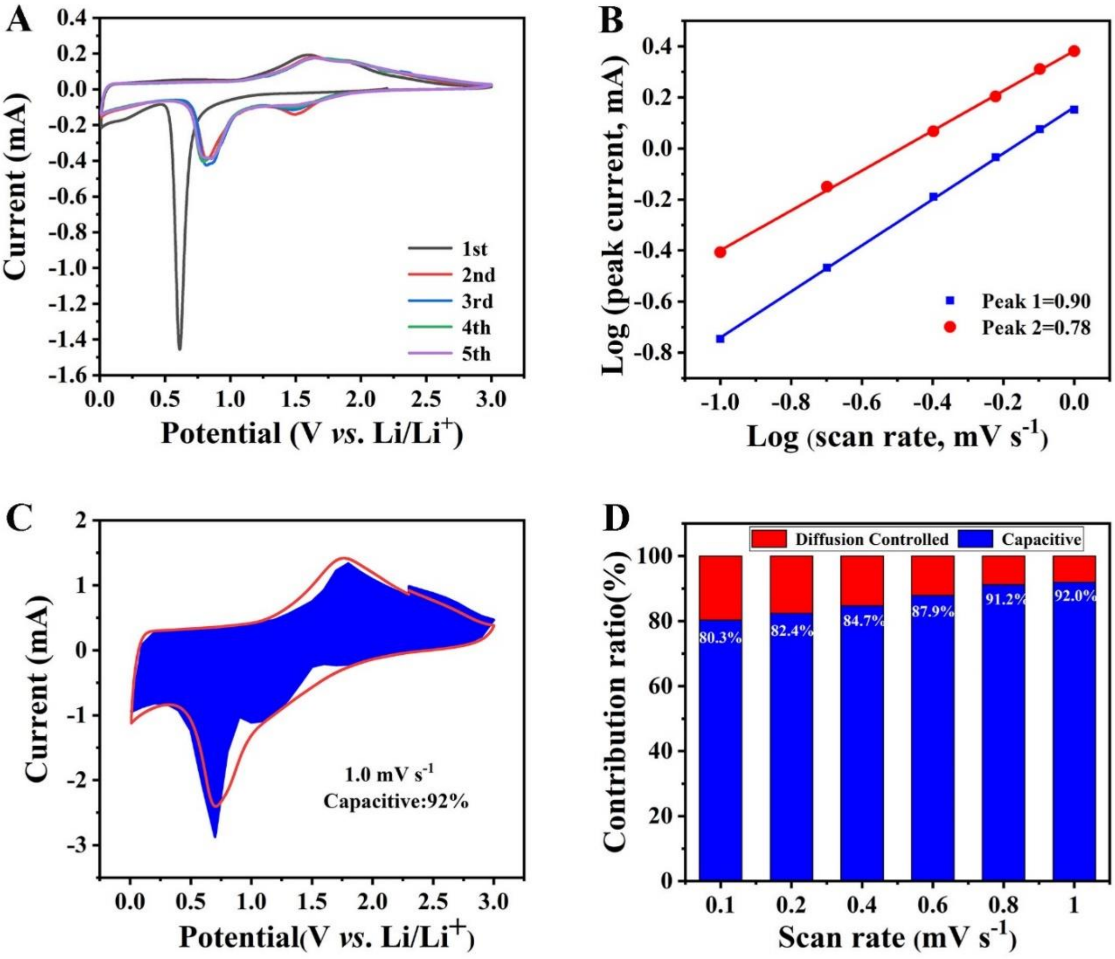


Figure S3. (A) Cyclic voltammetry curves of $NiFe_2O_4$ in the first five cycles at 0.1 mV $s^{-1}$; (B) Linear relationship between log*i* and log*v*; (C) The capacitive contribution of the $NiFe_2O_4$ electrode at 1.0 mV $s^{-1}$; (D)Pseudocapacitive contribution ratio at various scan rates.

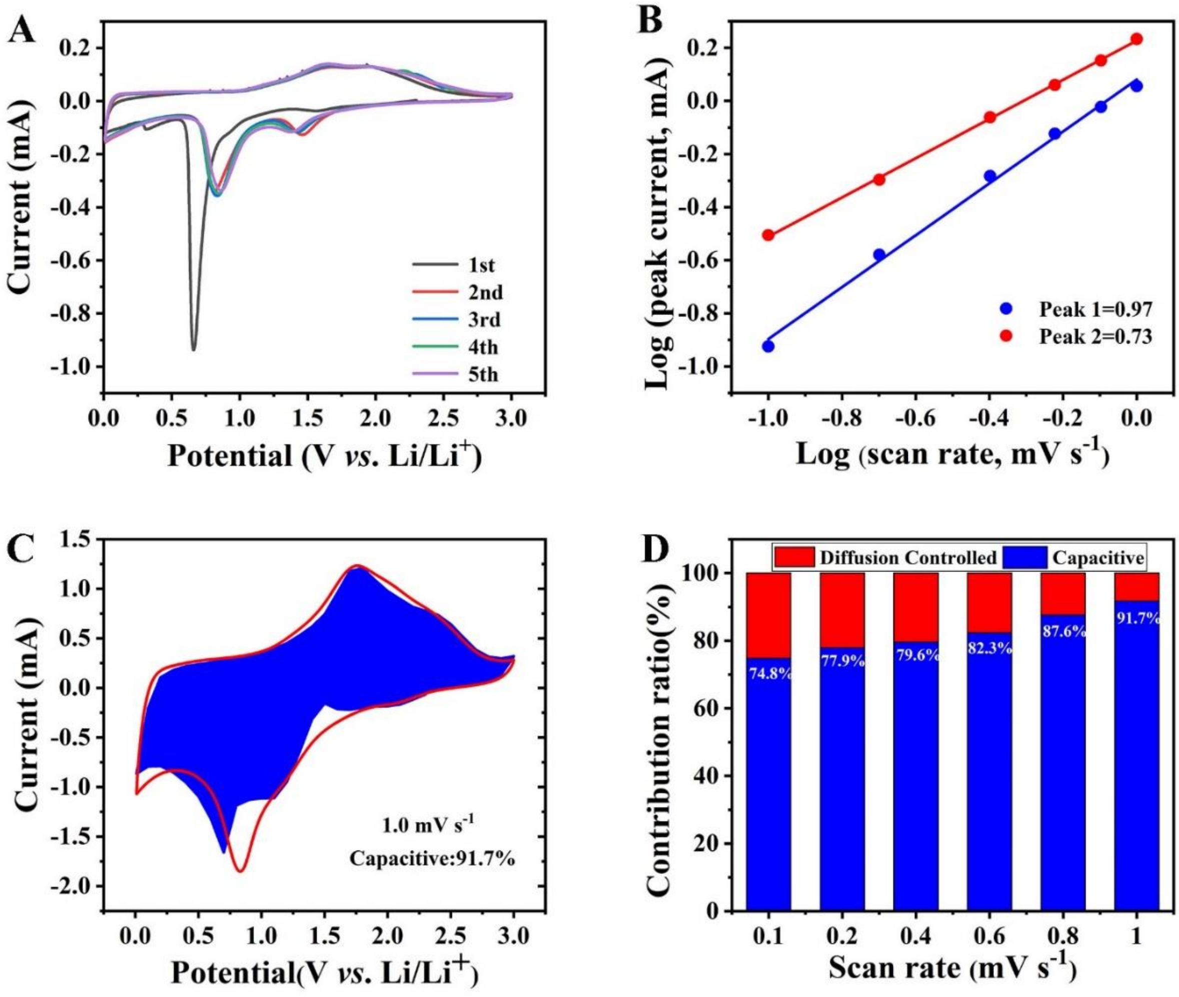


Figure S4. (A) Cyclic voltammetry curves of $CoFe_2O_4$ in the first five cycles at 0.1 mV $s^{-1}$; (B) Linear relationship between log*i* and log*v*; (C) The capacitive contribution of the $CoFe_2O_4$ electrode at 1.0 mV $s^{-1}$; (D)Pseudocapacitive contribution ratio at various scan rates.

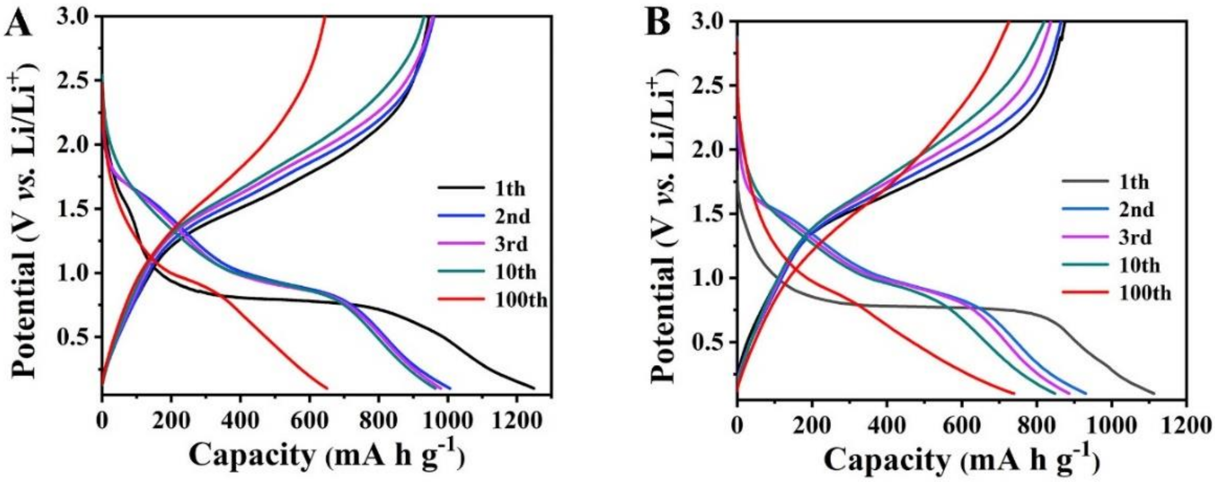


Figure S5. Discharge-charge curves of (A) $NiFe_2O_4$ and (B) $CoFe_2O_4$ at 200 mA $g^{-1}$.

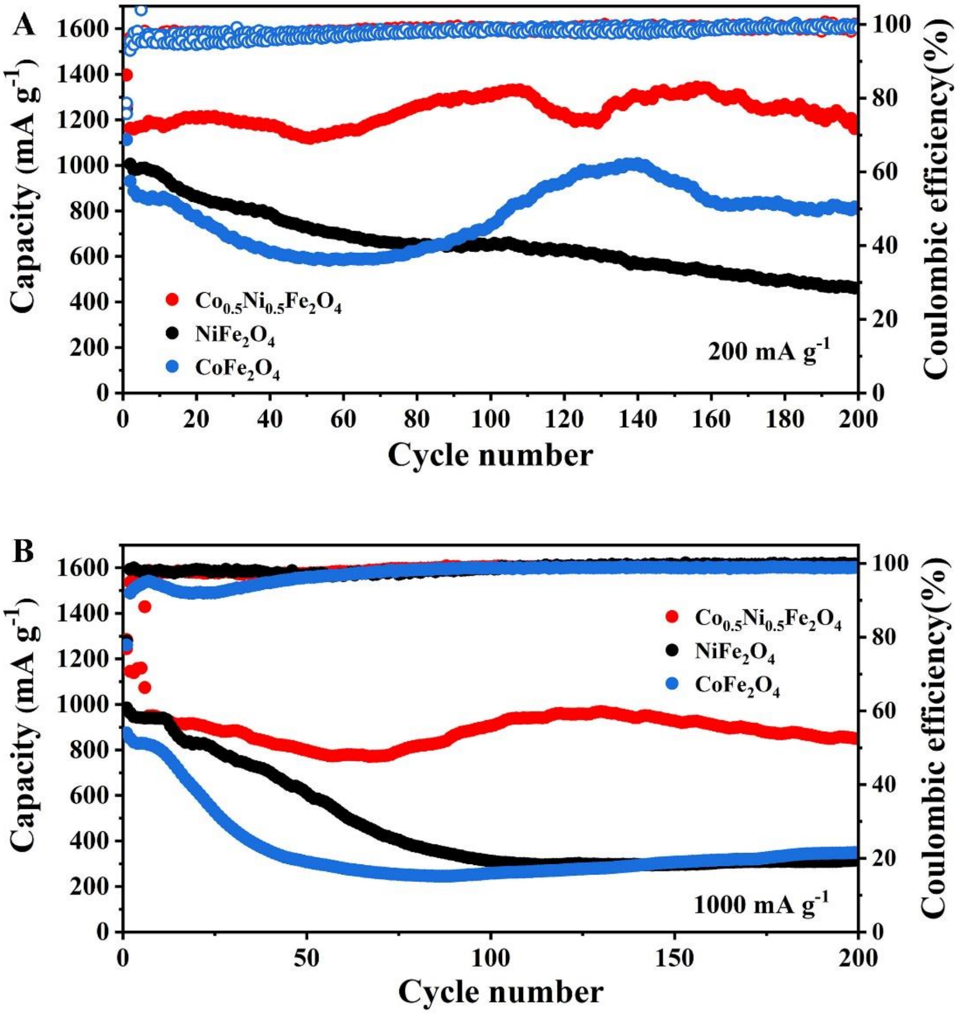


Figure S6. Cycling performance and CE of $Co_{0.5}Ni_{0.5}Fe_2O_4$, $NiFe_2O_4$ and $CoFe_2O_4$ for 200 cycles at(A) 200 mA $g^{-1}$and (B)1000 mA $g^{-1}$.
